# Existence of Multiple Solutions for a *p*-Kirchhoff Problem with Nonlinear Boundary Conditions

**DOI:** 10.1155/2013/516093

**Published:** 2013-07-25

**Authors:** Zonghu Xiu, Caisheng Chen

**Affiliations:** ^1^Science and Information College, Qingdao Agricultural University, Qingdao 266109, China; ^2^College of Science, Hohai University, Nanjing 210098, China

## Abstract

The paper considers the existence of multiple solutions of the singular nonlocal elliptic problem −*M*(∫_Ω_‍ | *x*|^−*ap*^ | ∇*u*|^*p*^)div(|*x*|^−*ap*^ | ∇*u*|^*p*−2^∇*u*) = *λh*(*x*) | *u*|^*r*−2^ *u*, *x* ∈ Ω, *M*(∫_Ω_‍ | *x*|^−*ap*^ | ∇*u*|^*p*^) | *x*|^−*ap*^ | ∇*u*|^*p*−2^ (∂*u*/∂*ν)* = *g*(*x*) | *u*|^*q*−2^ *u*, on ∂Ω, where 1 < (*N* + 1)/2 < *p* < *N*, *a* < (*N* − *p*)/*p*. By the variational method on the Nehari manifold, we prove that the problem has at least two positive solutions when some conditions are satisfied.

## 1. Introduction and Main Result 

 In this paper, we consider the existence of multiple solutions for the singular elliptic problem:

(1) −M(∫Ω|x|−ap|∇u|pdx)div⁡(|x|−ap|∇u|p−2∇u)    =λh(x)|u|r−2u, x∈Ω,M(∫Ω|x|−ap|∇u|p)|x|−ap|∇u|p−2∂u∂ν  =g(x)|u|q−2u, on⁡  ∂Ω,

where 1 < (*N* + 1)/2 < *p* < *N*, *a* < (*N* − *p*)/*p*, *λ* > 0, *Ω* is an exterior domain of ℝ^*N*^: that is, and *Ω* = ℝ^*N*^∖*D*, where *D* is a bounded domain in ℝ^*N*^ with the smooth boundary ∂*D*(  = ∂*Ω*), and 0 ∈ *Ω*. *g*(*x*) and *h*(*x*) are continuous functions, *M*(*s*) = *αs* + *β* with the parameters *α*, *β* > 0.

Problem like (1) is usually called nonlocal problem because of the presence of the integral over the entire domain, and this implies that (1) is no longer a pointwise identity. In fact, such kind of problem can be traced back to the work of Kirchhoff. In [1], Kirchhoff investigated the model of the form

(2) $$\begin{array}{c} \rho \frac{\partial^{2}u}{\partial t^{2}}-\left(\frac{P_{0}}{h}+\frac{E}{2L}\overset{L}{\underset{0}{\int }}{\left|\frac{\partial u}{\partial x}\right|}^{2}dx\right)\frac{\partial^{2}u}{\partial x^{2}}=0, \end{array}$$

where *ρ*, *P*_0_, *h*, *E*, and *L* are all positive constants. This equation extends the classical d'Alembert's wave equation by considering the effects of changes in the length of the strings during the vibrations. Problem (1) is related to the stationary analogue of problem (2). After Kirchhoff's work, various models of Kirchhoff-type have been studied by many authors: we refer the readers to [2–9]. In [4], by the variational methods, Bensedik and Bouchekif considered the problem

(3) $$\begin{array}{c} -M\left(\int_{\Omega }{\left|\nabla u\right|}^{2}dx\right)\Delta u=f\left(x,u\right),\;x\in \Omega ,\\ u=0,\;\mathrm{on}\;\;\partial \Omega , \end{array}$$

where *Ω* is a bounded domain in ℝ^*N*^. One of the assumptions made on *f*(*x*, *t*) in (3) is that (**f**_1_)  *f*(*x*, *t*) is continuous function on $\overset{-}{\Omega }\times \mathbb{R}$ such that

(4) $$\begin{array}{c} f\left(x,t\right)\;\geq 0,\;\forall t\geq 0,\;\;x\;\;\in \overset{-}{\Omega },\\ f\left(x,t\right)=0,\;\forall t\leq 0,\;\;x\in \overset{-}{\Omega }. \end{array}$$

The authors proved that problem (3) has a positive solution or has no solution when some other assumptions are fulfilled. In our paper, however, the weight functions *h*(*x*) and *g*(*x*) are permitted to change sign. Thus, the methods in [4] cannot be directly applied on (1).

In recent years, some other authors considered the Kirchhoff-type equations with *p*-Laplacian [10–13]. In fact, motivated by [4, 5] and our previous work [14], we consider the existence of multiple solutions for problem (1) on the Nehari manifold by variational methods. We prove that problem (1) has at least two positive solutions. Since *Ω* ⊂ ℝ^*N*^ is an unbounded domain and the problem is singular, the loss of compactness of the Sobolev embedding renders variational technique more delicate.

In order to state our result, we introduce a weighted Sobolev space *E* = *W*_*a*_^1,*p*^(*Ω*), which is the completion of the space *C*_0_^*∞*^(*Ω*) with the norm of

(5) $$\begin{array}{c} {||u||}_{E}={\left(\int_{\Omega }{\left|x\right|}^{-ap}{\left|\nabla u\right|}^{p}dx\right)}^{1/p}. \end{array}$$

For *ρ* ≥ 1 and *f* = *f*(*x*) > 0 in *Ω*, we define the space *L* ^*ρ*^(*Ω*, *f*) as being the set of Lebesgue measurable functions *u* : *Ω* → ℝ^1^, which satisfies

(6) $$\begin{array}{c} {||u||}_{L^{\rho }(\Omega ,f)}={\left(\int_{\Omega }f\left(x\right){\left|u\right|}^{\rho }dx\right)}^{1/\rho }<\infty . \end{array}$$

The following weighted Sobolev-Hardy inequality is due to Caffarelli et al. [15], which is called the Caffarelli-Kohn-Nirenberg inequality. There is a constant *S*_1_ > 0 such that

(7) (∫ℝN|x|−bp∗|u|p∗dx)1/p∗  ≤S1(∫ℝN|x|−ap|∇u|pdx)1/p, ∀u∈C0∞(ℝN),

where −*∞* < *a* < (*N* − *p*)/*p*, *a* ≤ *b* < *a* + 1, *d* = *a* + 1 − *b*, and p* = *pN*/(*N* − *pd*). Throughout this paper, we make the following assumptions:

 (*A*_1_)  *h*(*x*) | *x*|^*br*^ ∈ *L* ^*η*^(*Ω*)∩*L* ^*∞*^(*Ω*) with *η* = p*/(p* − *r*), *h* ^±^ = max⁡{±*h*(*x*), 0}≢0,
 (*A*_2_)  *g*(*x*) ∈ *C*(∂*Ω*)∩*L* ^*∞*^(∂*Ω*),
 (*A*_3_)  1 < *r* < *p* < 2*p* ≤ *q* < *p*_∗_ = *p*(*N* − 1)/(*N* − *p*).

Now, we give the definition of weak solution for problem (1).

**Definition**

A function *u* ∈ *E* is said to be a weak solution of problem (1) if for any *φ* ∈ *C*_0_^*∞*^(*Ω*)

(8) (α||u||Ep+β)∫Ω|x|−ap|∇u|p−2∇u·∇φdx   −∫∂Ωg(x)|u|q−2uφdσ  =λ∫Ωh(x)|u|r−2uφdx.

The assumptions (*A*_1_)–(*A*_3_) mean that all the integrals in (8) are well defined and convergent.

In view of (*A*_3_), it follows from the compact trace embedding *W*_*a*_^1,*p*^(*Ω*)↪*L* ^*q*^(∂*Ω*) [16] that

(9) $$\begin{array}{c} \int_{\partial \Omega }g\left(x\right){\left|u\right|}^{q}d\sigma \leq S_{2}{||g||}_{\infty }{||u||}_{E}^{q}, \end{array}$$

for some constant *S*_2_ > 0, and ||*g*||_*∞*_≜||*g*||_*L*^*∞*^(∂*Ω*)_.

Our main result is in the following.

**Theorem 2**

Assume (*A*_1_)–(*A*_3_); there exists λ* > 0 such that problem (1) has at least two positive solutions for all *λ* > 0 satisfying 0 < *λ* < λ*.

This paper is organized as follows. In Section 2, we give some properties of the Nehari manifold and set up the variational framework for problem (1). In Section 3, we consider the multiplicity results and prove Theorem 2.

## 2. Preliminary Results

 It is clear that problem (1) has a variational structure. Let *J*_*λ*_(*u*) : *E* → ℝ^1^ be the corresponding Euler functional of problem (1), which is defined by

(10) Jλ(u)=1pM^(||u||Ep)−1q∫∂Ωg(x)|u|qdσ−1r∫Ωh(x)|u|rdx,

where $\hat{M}(t)=\int_{0}^{t}M(s)ds$. Then, we see that the functional *J*_*λ*_(*u*) ∈ *C* ^1^(*E*, ℝ^1^), and for ∀*φ* ∈ *E*, there holds

(11) 〈Jλ′(u),φ〉=M(||u||Ep)∫Ω|x|−ap|∇u|p−2∇u·∇φdx−∫∂Ωg|u|q−2uφdσ−λ∫Ωh|u|r−2uφdx.

Particularly, we have

(12) 〈Jλ′(u),u〉=M(||u||Ep)∫Ω|x|−ap|∇u|pdx−∫∂Ωg(x)|u|qdσ−λ∫Ωh(x)|u|rdx.

It is well known that the weak solution of problem (1) is the critical point of *J*_*λ*_(*u*). Thus, to prove the existence of weak solutions for problem (1), it is sufficient to show that *J*_*λ*_(*u*) admits a sequence of critical points. Since *J*_*λ*_(*u*) is not bounded below on *E*, it is useful to consider the functional *J*_*λ*_(*u*) on the Nehari manifold [17, 18]:

(13) $$\begin{array}{c} N_{\lambda }=\left\{u\in E\setminus \left\{0\right\}\;|\;\langle J_{\lambda }^{\prime }\left(u\right),u\rangle =0\right\}, \end{array}$$

where 〈, 〉 denotes the usual duality. Then, it follows from (12) that *u* ∈ *N*_*λ*_ if and only if

(14) $$\begin{array}{c} M\left({||u||}_{E}^{p}\right){||u||}_{E}^{p}-\int_{\partial \Omega }g\left(x\right){\left|u\right|}^{q}d\sigma -\lambda \int_{\Omega }h\left(x\right){\left|u\right|}^{r}dx=0. \end{array}$$

Then, we get from (10)–(14) that

(15) Jλ(u)=(12p−1r)α||u||E2p−(1r−1p)β||u||Ep+(1r−1q)∫∂Ωg(x)|u|qdσ

(16) =(12p−1q)α||u||E2p+(1p−1q)β||u||Ep−(1r−1q)λ∫Ωh(x)|u|rdx.

We define

(17) $$\begin{array}{c} \Phi_{\lambda }\left(u\right)=\langle J_{\lambda }^{\prime }\left(u\right),u\rangle . \end{array}$$

Then, (14) implies that

(18) 〈Φλ′(u),u〉=pM′(||u||Ep)||u||E2p+pM(||u||Ep)||u||Ep−q∫∂Ωg(x)|u|qdσ−rλ∫Ωh(x)|u|rdx

(19) =(2p−q)α||u||E2p+(p−q)β||u||Ep+(q−r)λ∫Ωh(x)|u|rdx

(20) =(2p−r)α||u||E2p+(p−r)β||u||Ep−(q−r)∫∂Ωg(x)|u|qdσ.

It is natural to split *N*_*λ*_ into three parts:

(21) $$\begin{array}{c} N_{\lambda }^{+}=\left\{u\in N_{\lambda }\;|\;\langle \Phi_{\lambda }^{\prime }\left(u\right),u\rangle >0\right\},\\ N_{\lambda }^{-}=\left\{u\in N_{\lambda }\;|\;\langle \Phi_{\lambda }^{\prime }\left(u\right),u\rangle <0\right\},\\ N_{\lambda }^{0}=\left\{u\in N_{\lambda }\;|\;\langle \Phi_{\lambda }^{\prime }\left(u\right),u\rangle =0\right\}. \end{array}$$

Now, we give some important properties of *N*_*λ*_^+^, *N*_*λ*_^−^, and *N*_*λ*_^0^.

**Lemma 3**

Let 1 < *r* < *p* < *q* and *q* ≥ 2*p*. Then, *J*_*λ*_(*u*) is coercive and bounded below on *N*_*λ*_.

**Proof**

For 1 < *r* < *p* < *q*, we obtain from (*A*_1_), the Hölder and Caffarelli-Kohn-Nirenberg inequalities that

(22) ∫Ωh(x)|u|rdx≤(∫Ω(|h(x)||x|br)ηdx)1/η×(∫Ω|x|−bp∗|u|p∗dx)r/p∗≤S1rhη(∫Ω|x|−ap|∇u|pdx)r/p≤S1rhη||u||Er,

where *h*_*η*_ = (∫_*Ω*_(|*g*(*x*)||*x*|^*br*^)^*η*^ *dx*)^1/*η*^, *η* = p*/(p* − *r*). Thus, we get from (16) that

(23) Jλ(u)=(12p−1q)α||u||E2p+(1p−1q)β||u||Ep−(1r−1q)∫Ωh(x)|u|rdx≥||u||Ep[(12p−1q)α||u||Ep+(1p−1q)β]−(1r−1q)λS1rhη||u||Er.

Then, one can obtain by the Young inequality and *q* ≥ 2*p* that *J*_*λ*_(*u*) is coercive and bounded below on *N*_*λ*_.

Let

(24) $$\begin{array}{c} \lambda_{0}=\{\begin{array}{cc} \frac{2\sqrt{\left(q-2p\right)\left(q-p\right)}}{\left(q-r\right)S_{2}^{r}h_{\eta }} & \\ \;\times {\left[\frac{2\sqrt{\alpha \beta \left(2p-r\right)\left(p-r\right)}}{\left(q-r\right){||g||}_{\infty }}\right]}^{\left(3p-2r)/(2q-3p\right)}, & q>2p,\\ \frac{p\beta }{\left(2p-r\right)S_{1}^{r}h_{\eta }} & \\ \;\times {\left[\frac{2\sqrt{\alpha \beta \left(2p-r\right)\left(p-r\right)}}{\left(2p-r\right){||g||}_{\infty }}\right]}^{2(p-r)/p}, & q=2p. \end{array} \end{array}$$

Then, we have the following result.

**Lemma 4**

Let *r* < *p* < *q*, *q* ≥ 2*p*. Then, *N*_*λ*_^0^ = *∅* for 0 < *λ* < *λ*_0_.

**Proof**

Suppose that there exists *u* ∈ *N*_*λ*_^0^. If *q* > 2*p*, then it follows from (19) and (21) that

(25) 2β(q−2p)(q−p)||u||E3p/2  ≤(q−2p)α||u||E2p+β(q−p)||u||Ep  =(q−r)λ∫Ωh(x)|u|rdx  ≤(q−r)λS1rhη||u||Er,

which implies that

(26) $$\begin{array}{c} {||u||}_{E}\leq {\left[\frac{\left(q-r\right)\lambda S_{1}^{r}h_{\eta }}{2\sqrt{\left(q-2p\right)\left(q-p\right)}}\right]}^{2/(3p-2r)}. \end{array}$$

On the other hand, we can similarly get from (20) and (21) that

(27) 2αβ(2p−r)(p−r)||u||E3p/2  ≤(2p−r)α||u||E2p+(p−r)||u||Ep  =(q−r)∫∂Ωg(x)|u|qdσ  ≤(q−r)||g||∞||u||Eq,

which yields that

(28) $$\begin{array}{c} {||u||}_{E}\geq {\left[\frac{2\sqrt{\alpha \beta \left(2p-r\right)\left(p-r\right)}}{\left(q-r\right){||g||}_{\infty }}\right]}^{2/(2q-3p)}. \end{array}$$

Thus, inequalities (26) and (28) show that *λ* ≥ *λ*_0_, which contradicts the hypothesis of *λ*.

For *q* = 2*p*, we can similarly obtain from (19) and (21) that

(29) $$\begin{array}{c} {||u||}_{E}\leq {\left[\frac{\left(q-r\right)\lambda S_{1}^{r}h_{\eta }}{p\beta }\right]}^{1/(p-r)}. \end{array}$$

Thus, (28) and (29) imply that *λ* > *λ*_0_, which is also a contradiction. Therefore, we complete the proof.

**Lemma 5**

If *u*_0_ is a local minimizer of *J*_*λ*_(*u*) on *N*_*λ*_ and *u*_0_ ∉ *N*_*λ*_^0^, then *u*_0_ is a critical point of *J*_*λ*_(*u*).

**Proof**

Let

(30) F(u)=M(||u||Ep)||u||Ep−∫∂Ωg(x)|u|qdσ−λ∫Ωh(x)|u|rdx, ∀u∈E.

Consider the following minimizing problem:

(31) $$\begin{array}{c} \underset{u\in N_{\lambda }}{\min}\;J_{\lambda }\left(u\right),\;\text{subject}\;\;\text{to}\;\;F\left(u\right)=0. \end{array}$$

By the Lagrange multiplier principle, there exists *γ* ∈ ℝ^1^ such that

(32) $$\begin{array}{c} J_{\lambda }^{\prime }\left(u_{0}\right)=\gamma F^{\prime }\left(u_{0}\right), \end{array}$$

(33) $$\begin{array}{c} \langle J_{\lambda }^{\prime }\left(u_{0}\right),u_{0}\rangle =\gamma \langle F^{\prime }\left(u_{0}\right),u_{0}\rangle =\gamma \langle \Phi_{\lambda }^{\prime }\left(u_{0}\right),u_{0}\rangle . \end{array}$$

Since *u*_0_ ∈ *N*_*λ*_ and *u*_0_ ∉ *N*_*λ*_^0^, it follows from (33) that *γ* = 0; furthermore, *J*_*λ*_′(*u*_0_) = 0.

Now, we write *N*_*λ*_ = *N*_*λ*_^+^ ∪ *N*_*λ*_^−^ for 0 < *λ* < *λ*_0_ and define

(34) $$\begin{array}{c} \delta_{\lambda }^{+}=\underset{u\in N_{\lambda }^{+}}{\inf}\;J_{\lambda }\left(u\right),\;\;\delta_{\lambda }^{-}=\underset{u\in N_{\lambda }^{-}}{\inf}\;J_{\lambda }\left(u\right). \end{array}$$

Denote

(35) $$\begin{array}{c} \lambda_{1}=\frac{r\left(q-p\right)\beta }{p\left(q-r\right)S_{1}^{r}h_{\eta }}{\left[\frac{\beta \left(p-r\right)}{S_{2}\left(q-r\right){||g||}_{\infty }}\right]}^{\left(p-r)/(q-p\right)}. \end{array}$$

Then, the following results on *δ*_*λ*_^+^ and *δ*_*λ*_^−^ are established.

**Lemma 6**

Let *r* < *p* < *q*, *q* ≥ 2*p*, and 0 < *λ* < *λ*_1_; then

- *δ*_*λ*_^+^ < 0,
- there exists constant *k*_0_ > 0 such that *δ*_*λ*_^−^ > *k*_0_.

**Proof**

(i) For ∀*u* ∈ *N*_*λ*_^+^, we have from (19) and (21) that

(36) (q−2p)α2pq||u||E2p+(q−p)βpq||u||Ep  ≤(q−r)λpq∫∂Ωg(x)|u|qdσ,λ∫∂Ωg(x)|u|qdσ≥(q−p)βq−r||u||Ep.

Thus, (36) and (16) give that

(37)   Jλ(u)<(q−rpq−q−rqr)λ∫Ωh(x)|u|rdx≤−β(p−r)(q−p)qpr||u||Ep<0,

which implies that

(38) $$\begin{array}{c} \delta_{\lambda }^{+}=\underset{u\in N_{\lambda }^{+}}{\inf}\;J_{\lambda }\left(u\right)<0. \end{array}$$

(ii) Let *u* ∈ *N*_*λ*_^+^; one can deduce from (20) and (21) that

(39) $$\begin{array}{c} {||u||}_{E}\geq {\left[\frac{(p-r)\beta }{S_{2}(q-r){||g||}_{\infty }}\right]}^{1/(q-p)}. \end{array}$$

On the other hand, we obtain from (16), (26), and (39) that

(40) Jλ(u)≥(12p−1q)α||u||E2p+(1p−1q)β||u||Ep−(q−r)λrqS1rhη||u||Er≥||u||Er[(q−p)βpq||u||Ep−r−(q−r)λS1rhηqr]≥||u||Er[(q−p)βpq((p−r)βS2(q−r)||g||∞)(p−r)/(q−p)    −(q−r)λS1rhηrq].

Therefore, if 0 < *λ* < *λ*_1_, there exists *k*_0_ > 0 such that *δ*_*λ*_^−^ > *k*_0_.

For each *u* ∈ *E* with ∫_∂*Ω*_ *g*(*x*) | *u*|^*q*^ *dσ* > 0, we define

(41) $$\begin{array}{c} m_{\alpha }\left(t\right)=\alpha t^{2p-r}{||u||}_{E}^{2p}+\beta t^{p-r}{||u||}_{E}^{p}-t^{q-r}\int_{\partial \Omega }g{\left|u\right|}^{q}d\sigma . \end{array}$$

Then, *m*_*α*_(0) = 0, *m*_*α*_(*t*)→−*∞* as *t* → +*∞*, and *m*_*α*_(*t*) gets its unique maximum at the critical point *t*_*α*,max⁡_. Particularly, *m*_0_(*t*) gets its unique maximum at

(42) $$\begin{array}{c} t_{0,\max}={\left[\frac{\beta \left(p-r\right){||u||}_{E}^{p}}{\left(q-r\right)\int_{\partial \Omega }g{\left|u\right|}^{q}d\sigma }\right]}^{1/(q-p)}, \end{array}$$

and we have the following results.

**Lemma 7**

Let *r* < *p* < *q* < *p*_∗_, *q* ≥ 2*p*, and 0 < *λ* < (*p*/*r*)*λ*_1_. For each *u* ∈ *E* with ∫_∂*Ω*_ *g*(*x*) | *u*|^*q*^ *dσ* > 0, one has the following:

(i) if ∫_*Ω*_ *h*(*x*) | *u*|^*r*^ *dx* ≤ 0, then there exists *t* ^−^ > *t*_*α*,max⁡_ such that *t* ^−^ *u* ∈ *N*_*λ*_^−^ and
  (43) $$\begin{array}{c} J_{\lambda }\left(t^{-}u\right)=\underset{t\geq 0}{\sup}\;J_{\lambda }\left(tu\right); \end{array}$$
(ii) if ∫_*Ω*_ *h*(*x*) | *u*|^*r*^ *dx* > 0, then there exists 0 < *t* ^+^ < *t*_*α*,max⁡_ < *t* ^−^ such that *t* ^+^ *u* ∈ *N*_*λ*_^+^,  *t* ^−^ *u* ∈ *N*_*λ*_^−^, and
  (44) $$\begin{array}{c} J_{\lambda }\left(t^{+}u\right)=\underset{0<t\leq t_{\alpha ,\max}}{\inf}J_{\lambda }\left(tu\right),\\ J_{\lambda }\left(t^{-}u\right)=\underset{t\geq t_{\alpha ,\max}}{\sup}J_{\lambda }\left(tu\right). \end{array}$$

**Proof**

(i) For ∀*u* ∈ *E*, we define

(45) ϕ0(t)=〈Jλ′(tu),tu〉=t2pα||u||E2p+tpβ||u||Ep−tq×∫∂Ωg(x)|u|qdσ−trλ∫Ωh(x)|u|rdx,

(46) ϕ1(t)=〈Φλ′(tu),tu〉=2pαt2p||u||E2p+tpβ||u||Ep−tq∫∂Ωg|u|qdσ−trλ∫Ωh(x)|u|rdx,

(47) ϕ2(t)=Jλ(tu)=α2pt2p||u||E2p+βptp||u||Ep−1qtq∫∂Ωg|u|qdσ−λrtr∫Ωh(x)|u|rdx.

Since ∫_*Ω*_ *h*(*x*) | *u*|^*r*^ *dx* ≤ 0, there exists unique *t* ^−^ > *t*_*α*,max⁡_ such that

(48) $$\begin{array}{c} m_{\alpha }^{\prime }\left(t^{-}\right)<0,\;\;m_{\alpha }\left(t^{-}\right)=\int_{\Omega }h\left(x\right){\left|u\right|}^{r}dx. \end{array}$$

Thus, *ϕ*_0_(*t* ^−^) = *t* ^*r*^(*m*_*α*_(*t* ^−^) − ∫_*Ω*_ *h*(*x*) | *u*|^*r*^ *dx*) = 0, which implies that *t* ^−^ *u* ∈ *N*_*λ*_. It follows from (20) that

(49) ϕ1(t−)=(2p−r)α(t−)2p||u||E2p+(p−r)β(t−)p||u||Ep−(q−r)(t−)q∫∂Ωg|u|qdσ=(t−)r+1mα′(t−)<0;

that is, *t* ^−^ *u* ∈ *N*_*λ*_^−^. By (47), we obtain that

(50) ϕ2′(t)=αt2p−1||u||E2p+βtp−1||u||Ep−tq−1∫∂Ωg|u|qdσ−λtr−1∫Ωh(x)|u|rdx=tr−1[mα(t)−λ∫Ωh(x)|u|rdx],

which shows that *ϕ*_2_(*t*) increases for *t* ∈ [0, *t* ^−^] and decreases for *t* ∈ [*t* ^−^, +*∞*). Therefore,

(51) $$\begin{array}{c} J_{\lambda }\left(t^{-}u\right)=\phi_{2}\left(t^{-}\right)=\underset{t\geq 0}{\sup}{\;J}_{\lambda }\left(tu\right). \end{array}$$

(ii) We firstly want to prove that 0 < ∫_*Ω*_ *h*(*x*) | *u*|^*r*^ *dx* ≤ *m*_*α*_(*t*_*α*,max⁡_). In fact,

(52)   m0(t0,max⁡)=β[β(p−r)||u||Ep(q−r)∫∂Ωg|u|qdσ](p−r)/(q−p)||u||Ep−[β(p−r)||u||Ep(q−r)∫∂Ωg|u|qdσ](q−r)/(q−p)×∫∂Ωg|u|qdσ=β(q−r)/(q−p)||u||Ep(q−r)/(q−p)(p−rq−r)(p−r)/(q−p)×(q−pq−r)[1||g||∞S2||u||Eq](p−r)/(q−p)≥β(q−r)/(q−p)||u||Er(q−pq−r)×[p−r(q−r)||g||∞](p−r)/(q−p).

Then, if 0 < *λ* < (*p*/*r*)*λ*_1_, we have that

(53) mα(0)=0<λ∫Ωh(x)|u|rdx≤β(q−r)/(q−p)||u||Er(q−pq−r)[p−r(q−r)||g||∞](p−r)/(q−p)≤m0(t0,max⁡)<mα(tα,max⁡).

Since ∫_*Ω*_ *h*(*x*) | *u*|^*r*^ *dx* > 0, there exists 0 < *t* ^+^ < *t*_*α*,max⁡_ < *t* ^−^ such that

(54) $$\begin{array}{c} m_{\alpha }\left(t^{+}\right)=m_{\alpha }\left(t^{-}\right)=\lambda \int_{\Omega }h\left(x\right){\left|u\right|}^{r}dx, \end{array}$$

and *m*_*α*_′(*t* ^+^) > 0, *m*_*α*_′(*t* ^−^) < 0. Similar to the proof of (i), we get that *t* ^+^ *u* ∈ *N*_*λ*_^+^, *t* ^−^ *u* ∈ *N*_*λ*_^−^. We can deduce from (50) that *ϕ*_2_(*t*) decreases for *t* ∈ [0, *t* ^+^] and increases for *t* ∈ [*t* ^+^, *t*_*α*,max⁡_). Therefore,

(55) $$\begin{array}{c} J_{\lambda }\left(t^{+}u\right)=\phi_{2}\left(t^{+}\right)=\underset{0\leq t\leq t_{\alpha ,\max}}{\inf}J_{\lambda }\left(tu\right). \end{array}$$

Similarly,

(56) $$\begin{array}{c} J_{\lambda }\left(t^{-}u\right)=\phi_{2}\left(t^{-}\right)=\underset{t\geq t_{\alpha ,\max}}{\sup}J_{\lambda }\left(tu\right). \end{array}$$

Then, we complete the proof.

For each *u* ∈ *E* with ∫_*Ω*_ *h*(*x*) | *u*|^*r*^ *dx* > 0, we define

(57) $$\begin{array}{c} {\overset{-}{m}}_{\alpha }\left(t\right)=\alpha t^{2p-q}{||u||}_{E}^{2p}+\beta t^{p-q}{||u||}_{E}^{p}-\lambda t^{r-q}\int_{\Omega }h\left(x\right){\left|u\right|}^{r}dx. \end{array}$$

Then, ${\overset{-}{m}}_{\alpha }(t)\to -\infty$ as *t* → 0^+^ and ${\overset{-}{m}}_{\alpha }(t)\to 0$ as *t* → *∞*. Furthermore, ${\overset{-}{m}}_{\alpha }(t)$ gets its unique maximum at some certain point ${\overset{-}{t}}_{\alpha ,\max}$. Particularly, ${\overset{-}{m}}_{0}(t)$ gets its unique maximum at the critical point

(58) $$\begin{array}{c} {\overset{-}{t}}_{0}={\overset{-}{t}}_{0,\max}\left(u\right)={\left[\frac{\lambda \left(q-r\right)\int_{\Omega }h\left(x\right){\left|u\right|}^{r}dx}{\beta \left(q-p\right){||u||}_{E}^{p}}\right]}^{1/(p-r)}, \end{array}$$

then we have the following results.

**Lemma 8**

Let 1 < *r* < *p* < *q*, *q* ≥ 2*p*, and 0 < *λ* < *pλ*_1_/*r*. For each *u* ∈ *E* with ∫_*Ω*_ *h*(*x*) | *u*|^*r*^ *dx* > 0 one has the following:

(i) if ∫_∂*Ω*_ *g* | *u*|^*q*^ *dσ* < 0, then there exists unique $0<t^{+}<{\overset{-}{t}}_{\alpha ,\max}$ such that *t* ^+^ *u* ∈ *N*_*λ*_^+^ and
  (59) $$\begin{array}{c} J_{\lambda }\left(t^{+}u\right)=\underset{t\geq 0}{\inf}\;J_{\lambda }\left(tu\right); \end{array}$$
(ii) if ∫_∂*Ω*_ *g* | *u*|^*q*^ *dσ* ≥ 0, then there exists $0<t^{+}<{\overset{-}{t}}_{\alpha ,\max}<t^{-}$ such that *t* ^+^ *u* ∈ *N*_*λ*_^+^, *t* ^−^ *u* ∈ *N*_*λ*_^−^, and
  (60) $$\begin{array}{c} J_{\lambda }\left(t^{+}u\right)=\underset{0<t\leq {\overset{-}{t}}_{\alpha ,\max}}{\inf}J_{\lambda }\left(tu\right),\;\;J_{\lambda }\left(t^{-}u\right)=\underset{t\geq {\overset{-}{t}}_{\alpha ,\max}}{\sup}J_{\lambda }\left(tu\right). \end{array}$$

**Proof**

(i) Since ∫_∂*Ω*_ *g* | *u*|^*q*^ *dσ* < 0, there exists unique $0<t^{+}<{\overset{-}{t}}_{\alpha ,\max}$ such that

(61) $$\begin{array}{c} {\overset{-}{m}}_{\alpha }^{\prime }\left(t^{+}\right)>0,\;\;{\overset{-}{m}}_{\alpha }\left(t^{+}\right)=\int_{\partial \Omega }g{\left|u\right|}^{q}d\sigma . \end{array}$$

Note that (45) can be rewritten as

(62) $$\begin{array}{c} \phi_{0}\left(t\right)=t^{q}\left[{\overset{-}{m}}_{\alpha }\left(t\right)-\int_{\partial \Omega }g{\left|u\right|}^{q}d\sigma \right]. \end{array}$$

Thus, (61) shows that *t* ^+^ *u* ∈ *N*_*λ*_. By virtue of (14) and (46), we get that

(63) ϕ1(t+)=〈Φλ′(t+u),t+u〉=(t+)q+1[(2p−q)α(t+)2p−q−1     +(p−q)β(t+)p−q−1||u||Ep     −λ(r−q)(t+)r−q−1∫Ωh(x)|u|rdx]=(t+)q+1m−α′(t+)>0,

which implies that *t* ^+^ *u* ∈ *N*_*λ*_^+^. We have from (47) that

(64) $$\begin{array}{c} \phi_{2}^{\prime }\left(t\right)=t^{q-1}\left[{\overset{-}{m}}_{\alpha }\left(t\right)-\int_{\partial \Omega }g{\left|u\right|}^{q}d\sigma \right]. \end{array}$$

Then, *ϕ*_2_(*t*) decreases for *t* ∈ [0, *t* ^+^] and increases for *t* ∈ [*t* ^+^, +*∞*); that is,

(65) $$\begin{array}{c} J_{\lambda }\left(t^{+}u\right)=\underset{t\geq 0}{\inf}{\;J}_{\lambda }\left(tu\right). \end{array}$$

(ii) We need also to prove that $0<\int_{\partial \Omega }g|u{|}^{q}d\sigma <{\overset{-}{m}}_{\alpha }(t)$. In fact, we have that

(66) m−α(tα,max⁡)≥m−0(t−0)  =β[λ(q−r)∫Ωh|u|rdxβ(q−p)||u||Ep](p−q)/(p−r)||u||E2p   −λ[λ(q−r)∫Ωh|u|rdxβ(q−p)||u||Ep](r−q)/(p−r)  =λ(p−q)/(p−r)β(q−r)/(p−r)||u||Ep(q−r)/(p−r)   ×(q−pq−r)(q−p)/(p−r)   ×(p−rq−r)(∫Ωh|u|rdx)(p−q)/(p−r)  ≥λ(p−q)/(p−r)β(q−r)/(p−r)(q−rp−r)   ×(q−p(q−r)S1rhη)(q−p)/(p−r)||u||Eq  ≥∫∂Ωg|u|qdσ≥0

for 0 < *λ* ≤ (*p*/*r*)*λ*_1_.

The rest of the proof is similar to that of (ii) in Lemma 7, and here we omit the proof.

**Lemma 9**

Assume (*A*_1_)–(*A*_3_). If *u*_*n*_⇀*u* in *E*, then there exists a subsequence of {*u*_*n*_}, still denoted by {*u*_*n*_}, such that

(67) $$\begin{array}{c} \int_{\Omega }h\left(x\right){\left|u_{n}\right|}^{r}dx\to \int_{\Omega }h\left(x\right){\left|u\right|}^{r}dx,\;\\ \int_{\partial \Omega }g\left(x\right){\left|u_{n}\right|}^{q}d\sigma \to \int_{\partial \Omega }g\left(x\right){\left|u\right|}^{q}d\sigma . \end{array}$$

**Proof**

By the assumption (*A*_2_), we have *h*(*x*) | *x*|^*br*^ ∈ *L* ^*η*^(*Ω*)∩*L* ^*∞*^(*Ω*), and then for any *ε* > 0, there exists *R*_*ε*_ > 0 large enough such that

(68) $$\begin{array}{c} \int_{B_{R_{\varepsilon }}^{c}}{\left(\left|h\left(x\right)\right|{\left|x\right|}^{br}\right)}^{\eta }dx\leq \varepsilon^{\eta }, \end{array}$$

where *B*_*R*_ = {*x* ∈ ℝ^*N*^ | |*x* | ≤*R*}, *B*_*R*_^*c*^ = {*x* ∈ ℝ^*N*^ | |*x* | >*R*} for *R* > 0.

The compact embedding theorem *W*_*a*_^1,*p*^(*Ω*)↪*L* ^*r*^(*Ω*, |*x*|^−*α*^) (Theorem 2.1 in [19]) implies that

(69) $$\begin{array}{c} u_{n}\to u\;\text{a.e}.\;\;\text{in}\;\;\Omega . \end{array}$$

Inequality (7) shows that {*u*_*n*_} is bounded in *L* ^p*^(ℝ^*N*^, |*x*|^*br*^), which implies that *u*_*n*_⇀*u* in *L* ^p*^(ℝ^*N*^, |*x*|^*br*^). Thus, we can obtain that

(70) $$\begin{array}{c} \int_{B_{R_{\varepsilon }}\setminus D}{\left|x\right|}^{-bp^{*}}{\left|u_{n}-u\right|}^{p^{*}}dx<\varepsilon^{p^{*}},\\ \int_{\Omega }{\left|x\right|}^{-bp^{*}}{\left|u_{n}\right|}^{p^{*}}dx\leq M^{p^{*}},\\ \int_{\Omega }{\left|x\right|}^{-bp^{*}}{\left|u\right|}^{p^{*}}dx\leq M^{p^{*}} \end{array}$$

for some *M* > 0. On the other hand, we get from the Höder inequality and (68)–(70) that

(71) ∫BRεch|un−u|rdx  ≤(∫BRεc(|h||x|br)ηdx)1/η   ×(∫BRεc|x|−bp∗|un−u|p∗dx)r/p^∗    ≤2rMrε,∫BRε∖Dh|un−u|rdx  ≤(∫BRε∖D(|h||x|br)ηdx)1/η   ×(∫BRε∖D|x|−bp∗|un−u|p∗dx)r/p^∗  ≤c0εr

for some constant *c*_0_ > 0 and large *n*.

Thus, (71) implies that

(72) $$\begin{array}{c} \int_{\Omega }h\left(x\right){\left|u_{n}-u\right|}^{r}dx\to 0\;\text{as}\;\;n\to \infty ; \end{array}$$

that is,

(73) $$\begin{array}{c} \int_{\Omega }h\left(x\right){\left|u_{n}\right|}^{r}dx\to \int_{\Omega }h\left(x\right){\left|u\right|}^{r}dx\;\text{as}\;\;n\to \infty . \end{array}$$

Since ∂*Ω* is compact and *g* ∈ *C*(∂*Ω*)∩*L* ^*∞*^(∂*Ω*), we obtain by the trace embedding theorem in [20] that

(74) $$\begin{array}{c} \lambda \int_{\partial \Omega }g\left(x\right){\left|u_{n}\right|}^{q}d\sigma \to \lambda \int_{\partial \Omega }g\left(x\right){\left|u\right|}^{q}d\sigma \;\text{as}\;\;n\to \infty . \end{array}$$

This concludes the proof.

## 3. Existence of Solutions

In this part, we will give the proof of the existence of nonnegative and nontrivial solutions. Before this, we need to prove the following two important lemmas.

**Lemma 10**

Assume (*A*_3_) and 0 < *λ* < (*p*/*r*)*λ*_1_. Then, the functional *J*_*λ*_(*u*) has a minimizer *u*_0_^+^ ∈ *N*_*λ*_^+^ and

- *J*_*λ*_(*u*_0_^+^) = *δ*_*λ*_^+^,
- *u*_0_^+^ is a positive weak solution of problem (1).

**Proof**

Since *J*_*λ*_(*u*) is bounded in *N*_*λ*_^+^, there exists a minimizing sequence of {*u*_*k*_} ∈ *N*_*λ*_^+^ such that

(75) $$\begin{array}{c} \underset{k\to \infty }{\lim}{\;J}_{\lambda }\left(u_{k}\right)=\underset{u\in N_{\lambda }^{+}}{\inf}{\;J}_{\lambda }\left(u\right). \end{array}$$

We can get that {*u*_*k*_} is bounded in *E* and *u*_*k*_⇀*u*_0_^+^ weakly in *E* since *J*_*λ*_(*u*) is coercive. It follows from Lemma 6 that

(76) $$\begin{array}{c} J_{\lambda }\left(u_{k}\right)\to \delta_{\lambda }^{+}<0\;\text{as}\;\;k\to +\infty . \end{array}$$

Furthermore, equality (16) and Lemma 9 imply that ∫_*Ω*_ *h*(*x*) | *u*_0_^+^|^*r*^ *dx* > 0. In the following, we prove that *u*_*k*_ → *u*_0_^+^ in *E*. Suppose otherwise that

(77) $$\begin{array}{c} {||u_{0}^{+}||}_{E}<\underset{k\to +\infty }{\lim}\inf{||u_{k}||}_{E}. \end{array}$$

Let

(78) mα,u0+(t)=αt2p−q||u0+||E2p+βtp−q||u0+||Ep−λtr−q∫Ωh(x)|u0+|rdx,φ0(t)=αt2p−q||u0+||E2p+βtp−q||u0+||Ep−λtr−q∫Ωh(x)|u0+|rdx−∫∂Ωg(x)|u0+|qdσ.

It is obvious that *m*_*α*,*u*_0_^+^_(*t*) has the unique critical point *t*_*α*,max⁡_(*u*_0_^+^) and *m*_*α*,*u*_0_^+^_(*t*) increases for *t* ∈ [0, *t*_*α*,max⁡_(*u*_0_^+^)] and decreases for *t* ∈ [*t*_*α*,max⁡_(*u*_0_^+^), *∞*). Particularly, *m*_0,*u*_0_^+^_(*t*) has the unique critical point at $t_{0}(u_{0}^{+})={\overset{-}{t}}_{0,\max}(u_{0}^{+})$. Since ∫_*Ω*_ *h*(*x*) | *u*_0_^+^|^*r*^ *dx* > 0, it follows from Lemma 8 that there exists *t*_0_^+^ < *t*_*α*,max⁡_(*u*_0_^+^) such that *t*_0_^+^ *u*_0_^+^ ∈ *N*_*λ*_^+^ and

(79) $$\begin{array}{c} J_{\lambda }\left(t_{0}^{+}u_{0}^{+}\right)=\underset{0\leq t\leq t_{\alpha ,\max}\left(u_{0}^{+}\right)}{\inf}J_{\lambda }\left(tu_{0}^{+}\right). \end{array}$$

By virtue of *t*_0_^+^ *u*_0_^+^ ∈ *N*_*λ*_^+^, we obtain that

(80) $$\begin{array}{c} \varphi_{0}\left(t_{0}^{+}\right)={\left(t_{0}^{+}\right)}^{-q}\langle J_{\lambda }\left(t_{0}^{+}u_{0}^{+}\right),t_{0}^{+}u_{0}^{+}\rangle =0. \end{array}$$

Similarly, we set

(81) φk(t)=αt2p−q||uk||E2p+βtp−q||uk||Ep−λtr−q∫Ωh(x)|uk|rdx−∫∂Ωg(x)|uk|qdσ.

In view of (77), we get that *φ*_*k*_(*t*_0_^+^) > *φ*_0_(*t*_0_^+^) = 0 for large *k*. Since *φ*_*k*_(1) = 0 and *φ*_*k*_′(*t*) ≥ *m*_*α*,*u*_0_^+^_′(*t*) > 0 for *t* ∈ (0, *t*_*α*,max⁡_(*u*_0_^+^)], we obtain that 1 < *t*_0_^+^ < *t*_*α*,max⁡_(*u*_0_^+^) and *φ*_*k*_(*t*) < 0 for *t* ∈ [0,1]. Then, by (77), (79), and Lemma 9, we get that

(82) $$\begin{array}{c} J_{\lambda }\left(t_{0}^{+}u_{0}^{+}\right)\leq J_{\lambda }\left(u_{0}^{+}\right)<\underset{k\to +\infty }{\lim}J_{\lambda }\left(u_{k}\right)=\delta_{\lambda }^{+}, \end{array}$$

which is a contradiction. Hence, *u*_*k*_ → *u*_0_^+^ strongly in *E*, and

(83) $$\begin{array}{c} J_{\lambda }\left(u_{k}\right)\to J_{\lambda }\left(u_{0}^{+}\right)=\delta_{\lambda }^{+}\;\text{as}\;\;k\to +\infty . \end{array}$$

Thus, *u*_0_^+^ is a minimum of *J*_*λ*_(*u*) on *N*_*λ*_^+^. Since *J*_*λ*_(*u*_0_^+^) = *J*_*λ*_(|*u*_0_^+^|) and |*u*_0_^+^ | ∈*N*_*λ*_^+^, we may assume by Lemma 5 that *u*_0_^+^ is a positive solution of problem (1).

**Lemma 11**

Assume (*A*_3_) and 0 < *λ* < (*p*/*r*)*λ*_1_. Then, the functional *J*_*λ*_(*u*) has a minimizer *u*_0_^−^ ∈ *N*_*λ*_^−^ and

- *J*_*λ*_(*u*_0_^−^) = *δ*_*λ*_^−^,
- *u*_0_^−^ is a positive weak solution of problem (1).

**Proof**

Since *J*_*λ*_(*u*) is bounded on *N*_*λ*_^−^, there exists a minimizing sequence {*u*_*k*_} ∈ *N*_*λ*_^−^ such that

(84) $$\begin{array}{c} \underset{k\to \infty }{\lim}\;J_{\lambda }\left(u_{k}\right)=\underset{u\in N_{\lambda }^{-}}{\inf}\;J_{\lambda }\left(u\right). \end{array}$$

Similar to the proof of Lemma 10, we may assume that *u*_*k*_⇀*u*_0_^−^ weakly in *E*. For *u*_*k*_ ∈ *N*_*λ*_^−^, we deduce by Lemma 6 and (15) that ∫_∂*Ω*_ *g*(*x*) | *u*_*k*_|^*q*^ *dσ* > 0; furthermore, ∫_∂*Ω*_ *g*(*x*) | *u*_0_^−^|^*q*^ *dσ* > 0. We also want to prove that *u*_*k*_ → *u*_0_^−^ strongly in *E*. In fact, if not, we have

(85) $$\begin{array}{c} {||u_{0}^{+}||}_{E}<\underset{k\to +\infty }{\lim}\inf{||u_{k}||}_{E}. \end{array}$$

By virtue of Lemma 7, there exists *t*_0_^−^ > 0 such that

(86) $$\begin{array}{c} J_{\lambda }\left(t_{0}^{-}u\right)=\underset{t\geq 0}{\sup}\;J_{\lambda }\left(tu_{k}\right), \end{array}$$

and a simple transformation shows that

(87) $$\begin{array}{c} J_{\lambda }\left(u_{k}\right)\geq J_{\lambda }\left(tu_{k}\right),\;\forall u_{k}\in N_{\lambda }^{-}. \end{array}$$

Therefore, Lemma 9 together with (84)–(87) gives that

(88) $$\begin{array}{c} J_{\lambda }\left(t_{0}^{-}u_{0}^{-}\right)<\underset{k\to +\infty }{\lim}J_{\lambda }\left(t_{0}^{-}u_{k}\right)\leq \underset{k\to +\infty }{\lim}J_{\lambda }\left(u_{k}\right)=\delta_{\lambda }^{-}, \end{array}$$

which is a contradiction, and we complete the proof.

**Proof of Theorem 2**

We set λ* = min⁡{*λ*_1_, *λ*_0_}. When 0 < *λ* < λ*, by Lemmas 10 and 11, we obtain that problem (1) has two nontrivial nonnegative solutions *u*_0_^+^ ∈ *N*_*λ*_^+^, *u*_0_^−^ ∈ *N*_*λ*_^−^.  Lemma 4 and the assumptions of Theorem 2 imply that *N*_*λ*_^+^∩*N*_*λ*_^−^ = *∅*; then *u*_0_^+^ and *u*_0_^−^ are distinct. Furthermore, since *J*_*λ*_(*u*_0_^±^) = *J*_*λ*_(|*u*_0_^±^|) and |*u*_0_^±^ | ∈*N*_*λ*_^±^, we can assume that the solutions *u*_0_^+^ and *u*_0_^−^ are positive. This completes the proof.

## 4. Conclusions

 The object of this paper is to prove the existence of multiple solutions for the nonlinear Kirchhoff-type problem (1). By the variational methods, we discuss the problem on the Nehari manifold and give the sufficient conditions for the existence of solutions. We overcome the difficulty due to the loss of compactness on the unbounded domain. In the future work, we are interested to consider similar problems, but the term on the right will be replaced by abstract functions.

## References

[1] Kirchhoff G (1883). *Mechanik*.

[2] Anello G (2011). A uniqueness result for a nonlocal equation of Kirchhoff type and some related open problem. *Journal of Mathematical Analysis and Applications*.

[3] Alves CO, Corrêa FJSA, Figueiredo GM (2010). On a class of nonlocal elliptic problems with critical growth. *Differential Equations & Applications*.

[4] Bensedik A, Bouchekif M (2009). On an elliptic equation of Kirchhoff-type with a potential asymptotically linear at infinity. *Mathematical and Computer Modelling*.

[5] Chen C-Y, Kuo Y-C, Wu T-F (2011). The Nehari manifold for a Kirchhoff type problem involving sign-changing weight functions. *Journal of Differential Equations*.

[6] Li YH, Li FY, Shi JP (2012). Existence of a posit solutions to Kirchhoff type problems without compactness conditons. *Journal of Differential Equations*.

[7] Perera K, Zhang Z (2006). Nontrivial solutions of Kirchhoff-type problems via the Yang index. *Journal of Differential Equations*.

[8] Wang F, An Y (2009). Existence of nontrivial solution for a nonlocal elliptic equation with nonlinear boundary condition. *Boundary Value Problems*.

[9] Zhang Z, Perera K (2006). Sign changing solutions of Kirchhoff type problems via invariant sets of descent flow. *Journal of Mathematical Analysis and Applications*.

[10] Cammaroto F, Vilasi L (2011). Multiple solutions for a Kirchhoff-type problem involving the *p*(*x*)-Laplacian operator. *Nonlinear Analysis: Theory, Methods and Applications*.

[11] Corrêa FJSA, Figueiredo GM (2009). On a *p*-Kirchhoff equation via Krasnoselskii’s genus. *Applied Mathematics Letters*.

[12] Dai G, Wei J (2010). Infinitely many non-negative solutions for a *p*(*x*)-Kirchhoff-type problem with Dirichlet boundary condition. *Nonlinear Analysis: Theory, Methods and Applications*.

[13] Ji C (2012). Infinitely many radial solutions for the *p*(*x*)-Kirchhoff-type equation with oscillatory nonlinearities in RN. *Journal of Mathematical Analysis and Applications*.

[14] Xiu ZH (2012). Existence of multiple solutions for a singular elliptic problem with critical sobolev expoent. *Abstract and Applied Analysis*.

[15] Caffarelli, Kohn R, Nirenberg L (1984). First order interpolation inequalities with weights. *Compositio Mathematica*.

[16] Díaz JI (1985). Nonlinear partial differential equations and free boundaries. *Elliptic Equations*.

[17] Brown KJ (2005). The Nehari manifold for a semilinear elliptic equation involving a sublinear term. *Calculus of Variations and Partial Differential Equations*.

[18] Nehari Z (1960). On a class of nonlinear second-order differential equations. *Transactions of the American Mathematical Society*.

[19] Xuan B (2005). The solvability of quasilinear Brezis-Nirenberg-type problems with singular weights. *Nonlinear Analysis: Theory, Methods and Applications*.

[20] Rodrigues RDS (2010). On elliptic problems involving critical Hardy-Sobolev exponents and sign-changing function. *Nonlinear Analysis: Theory, Methods and Applications*.

